# Electronic spin separation induced by nuclear motion near conical intersections

**DOI:** 10.1038/s41467-020-20831-8

**Published:** 2021-01-29

**Authors:** Yanze Wu, Joseph E. Subotnik

**Affiliations:** Department of Chemistry, University of Pennsylvania, Philadelphia, PA USA

**Keywords:** Reaction kinetics and dynamics, Reaction mechanisms

## Abstract

Though the concept of Berry force was proposed thirty years ago, little is known about the practical consequences of this force as far as chemical dynamics are concerned. Here, we report that when molecular dynamics pass near a conical intersection, a massive Berry force can appear as a result of even a small amount of spin-orbit coupling (<10^−3^ eV), and this Berry force can in turn dramatically change pathway selection. In particular, for a simple radical reaction with two outgoing reaction channels, an exact quantum scattering solution in two dimensions shows that the presence of a significant Berry force can sometimes lead to spin selectivity as large as 100%. Thus, this article opens the door for organic chemists to start designing spintronic devices that use nuclear motion and conical intersections (combined with standard spin-orbit coupling) in order to achieve spin selection. Vice versa, for physical chemists, this article also emphasizes that future semiclassical simulations of intersystem crossing (which have heretofore ignored Berry force) should be corrected to account for the spin polarization that inevitably arises when dynamics pass near conical intersections.

## Abstract (web summary)

Spin polarization is at the basis of quantum information and underlies some natural processes, but many aspects still need to be explored. Here, the authors, by quantum mechanical computations, show that even a weak spin-orbit coupling near a conical intersection can induce large spin selection, with consequences for spin manipulation in photochemical or electrochemical reactions.

## Introduction

Electronic spin is one of the most fundamental observables in quantum mechanics, and manipulating spin (so-called “spintronics”) is an enormous and exciting field of research today^1–7^. Even though the energy associated with flipping a single spin is incredibly small (6 μeV in the presence of a 0.1 T magnetic field), non-trivial spin manipulation can be achieved today through various techniques that center around the coupling between electronic motion and spin dynamics. Today, there are many physicists studying how giant and tunnel magnetoresistance^8,9^, spin–orbit torques^10–12^, spin–transfer torques^13–17^, and spin Hall effects^18–20^ can produce spin polarization either in the presence of external magnetic fields or carefully chosen solid-state materials with some degree of ferromagnetism or both. Interestingly, however, recent chiral-induced spin selectivity (CISS) experiments by Naaman, Waldeck, and co-workers^21–23^ have demonstrated that unusually large electronic spin polarization can also arise when current is passed through chiral molecules without ferromagnetic materials or magnetic fields (and despite very small spin–orbit coupling (SOC) matrix elements). It would appear that, as a community, we still have a great deal to learn about the subtle means by which non-trivial spin effects emerge in practice.

One means of achieving spin polarization that has not yet been fully explored theoretically (and one that appears to have large experimental consequences) is the coupling of molecular nuclear motion to electronic spin in the presence of SOC (but without any magnetic fields). In the simplest approximation, for a radical reaction with an odd number of electrons, one can consider a two-state Hamiltonian in a diabatic $\{\left|a\right\rangle ,\left|b\right\rangle \}$ basis of the form (where *T*_nu_ is the nuclear kinetic operator, *r*, *θ* are polar coordinates of nuclear position),

1 $${H}_{\text{tot}}={T}_{\text{nu}}+{H}_{\uparrow \uparrow }$$

2 $${H}_{\uparrow \uparrow }=\left[\begin{array}{ll}{E}_{a}(r,\theta )&V(r,\theta ){e}^{iWr}\\ V(r,\theta ){e}^{-iWr}&{E}_{b}(r,\theta )\end{array}\right]$$

Here *H*_↑↑_ represents the Hamiltonian with an extra up electronic spin and note that ${H}_{\downarrow \downarrow }={H}_{\uparrow \uparrow }^{* }$ because of time reversibility^24^. In Eq. (1), we assume that we can ignore the vectorial nature of the SOC and replace *H*_SOC_ = **L** ⋅ **S** ≈ *L*_*z*_*S*_*z*_. Without this assumption, we would necessarily need to include four electronic states and model the interaction between the up and down electronic states, i.e. *H*_↑↓_. Luckily, previous results^25^ suggest that the case of two states is usually not very different from the case of four states. Moreover, ref. ^25^ also demonstrates that nuclear dynamics on diabat $\left|a\right\rangle$ can lead to spin polarization on diabat $\left|b\right\rangle$ provided that (i) there is no spatial inversion between diabats $\left|a\right\rangle$ and $\left|b\right\rangle$, (ii) the nuclei are not in thermal equilibrium on diabat $\left|a\right\rangle$; (iii) the diabatic coupling (*V*(*r*, *θ*)*e*^*iWr*^) does not have a constant phase, i.e., *W* ≠ 0. In such a case, the relative difference in the state-to-state transmission rate between up and down electronic spin can be as large as 10% for a model system with reasonable parameters.

For the Hamiltonian in Eq. (1), the underlying physical mechanism behind any possible spin polarization here is the Lorentz-like force (**F**_B_) arising from the Berry curvature of the electronic surfaces^26–28^. This force is ignored by most dynamical simulation tools in chemistry^29^ and historically Berry forces have been presumed small in chemistry^30^; nevertheless, **F**_B_ can be incredibly important (as shown below). Let $\left|0\right\rangle$ and $\left|1\right\rangle$ be the ground and excited adiabatic electronic states of *H*_↑↑_. For a nuclear wavepacket moving along $\left|0\right\rangle$ with electronic spin up, the Berry force is equal and opposite to what a nuclear wavepacket with an electronic spin down in state ${\left|0\right\rangle }^{* }$ would feel moving along *H*_↓↓_^25^:

3 $${{\bf{F}}}_{{\rm{B}}}^{\uparrow }=\frac{2\hbar }{M}{\rm{Im}}\{{{\bf{d}}}_{01}({\bf{p}}\cdot {{\bf{d}}}_{10})\}=\frac{\hbar W}{M}\zeta (r,\theta )[{p}_{y},-{p}_{x}]=-{{\bf{F}}}_{{\rm{B}}}^{\downarrow }$$

4 $$\zeta (r,\theta )=\frac{1}{r}\frac{\partial }{\partial \theta }\left(\frac{{E}_{A}-{E}_{B}}{\sqrt{{({E}_{A}-{E}_{B})}^{2}+4{V}^{2}}}\right)$$

Here, **p** is the nuclear momentum, *M* is the nuclear mass, and ${{\bf{d}}}_{01}=\left\langle 0\right|{\boldsymbol{\nabla }}H\left|1\right\rangle /({E}_{1}-{E}_{0})$ is the derivative coupling between the two adiabats. As a consequence of Berry’s magnetic force, a nuclear wavepacket with one spin can follow an entirely different trajectory from an identical nuclear wavepacket with the opposite spin. According to Eqs. (3), (4), all spin separation will be proportional to the parameter *W* (which reflects the phase of the diabatic coupling) but inversely proportional to the parameter *V*.

Now if one wishes to map a realistic ab initio Hamiltonian to the reduced Hamiltonian in Eq. (1), one can roughly estimate the parameter *W* (which represents the gradient of the coupling phase) from the ratio of the SOC strength to diabatic coupling strength. In other words, for a two-state Hamiltonian *H* = *H*_0_ + *H*_SOC_, where *H*_0_ is the real-valued, standard electronic Hamiltonian and *H*_SOC_ is purely imaginary, the phase of the diabatic coupling $\left\langle a\right|H\left|b\right\rangle$ can be expressed as

5 $$\phi ={\tan }^{-1}\frac{\left|\left\langle a\right|{H}_{\text{SOC}}\left|b\right\rangle \right|}{\left\langle a\right|{H}_{0}\left|b\right\rangle }$$

When *H*_SOC_ ≪ *H*_0_, we have

6 $$W=\frac{\partial \phi }{\partial r}=\frac{\partial }{\partial r}{\tan }^{-1}\frac{\left|\left\langle a\right|{H}_{\text{SOC}}\left|b\right\rangle \right|}{\left\langle a\right|{H}_{0}\left|b\right\rangle }\approx \frac{\partial }{\partial r}\frac{\left|\left\langle a\right|{H}_{\text{SOC}}\left|b\right\rangle \right|}{\left\langle a\right|{H}_{0}\left|b\right\rangle }$$

Thus, for molecules or molecular assemblies, given that the SOC strengths are usually small ($\left|\left\langle a\right|{H}_{\text{SOC}}\left|b\right\rangle \right|$ < 1 meV) and diabatic couplings are usually much larger ($\left\langle a\right|{H}_{0}\left|b\right\rangle$ > 10 meV) in normal avoided crossings, one would not expect that *W* should be very large. And, as just mentioned, for a reasonably sized *W*, we do not expect >10% polarization (if at all).

However, the situation is more subtle around a conical intersection (CI)^31–33^, which is known to be essential for mediating a vast array of photochemical relaxation processes. On the one hand, the canonical thinking heretofore has always been that the geometric Berry force around a CI (as caused exclusively by a complex-valued Hamiltonian) should not lead to drastically different nuclear dynamics—a real-valued Hamiltonian should contain the bulk of nuclear dynamics through a CI. For example, in his seminal paper on geometric magnetism, Berry argued: “These classical effects will however be weak, since the monopole strength is $\pm\!\frac{1}{2}\hbar$, which vanishes in the classical limit. Moreover, the breakdown of the adiabatic approximation will be greatest at the degeneracies, because of transitions between adiabatic states”^28^. That being said, on the other hand, in the vicinity of a CI, the complex-valued diabatic coupling goes to zero $(\left\langle a\right|H\left|b\right\rangle \to 0)$ and the derivative coupling **d**_01_ diverges to infinity^34–37^. In such cases, even a tiny SOC can lead to a huge *W* and thus a huge magnetic force as estimated by Eq. (3). And so, despite Berry’s arguments in ref. ^28^ regarding nuclear dynamics through a CI, there is clearly a strong motivation to measure and quantify any nuclear dynamical spin polarization around or in the vicinity of a CI.

In this work, we follow the present train of thought and investigate coupled nuclear–spin dynamics around an “avoided” complex-valued CI. More precisely, our target system is a real-valued spin-free Hamiltonian (*H*_0_) for which we find a CI; however, we add to this Hamiltonian a second, constant complex-valued Hamiltonian (*H*_SOC_), which formally eliminates or moves away the CI^38–40^. We find that in this case the Berry magnetic force can yield a truly enormous effect, with spin-separation efficiencies close to 100% at certain energies. Furthermore, because the degree of spin polarization depends on the ratio between the SOC and the non-SOC diabatic coupling, and the diabatic coupling vanishes at a CI, we find that a huge amount of spin polarization can occur even with a very weak SOC.

## Results

### Model Hamiltonian

**Fig. 1:**
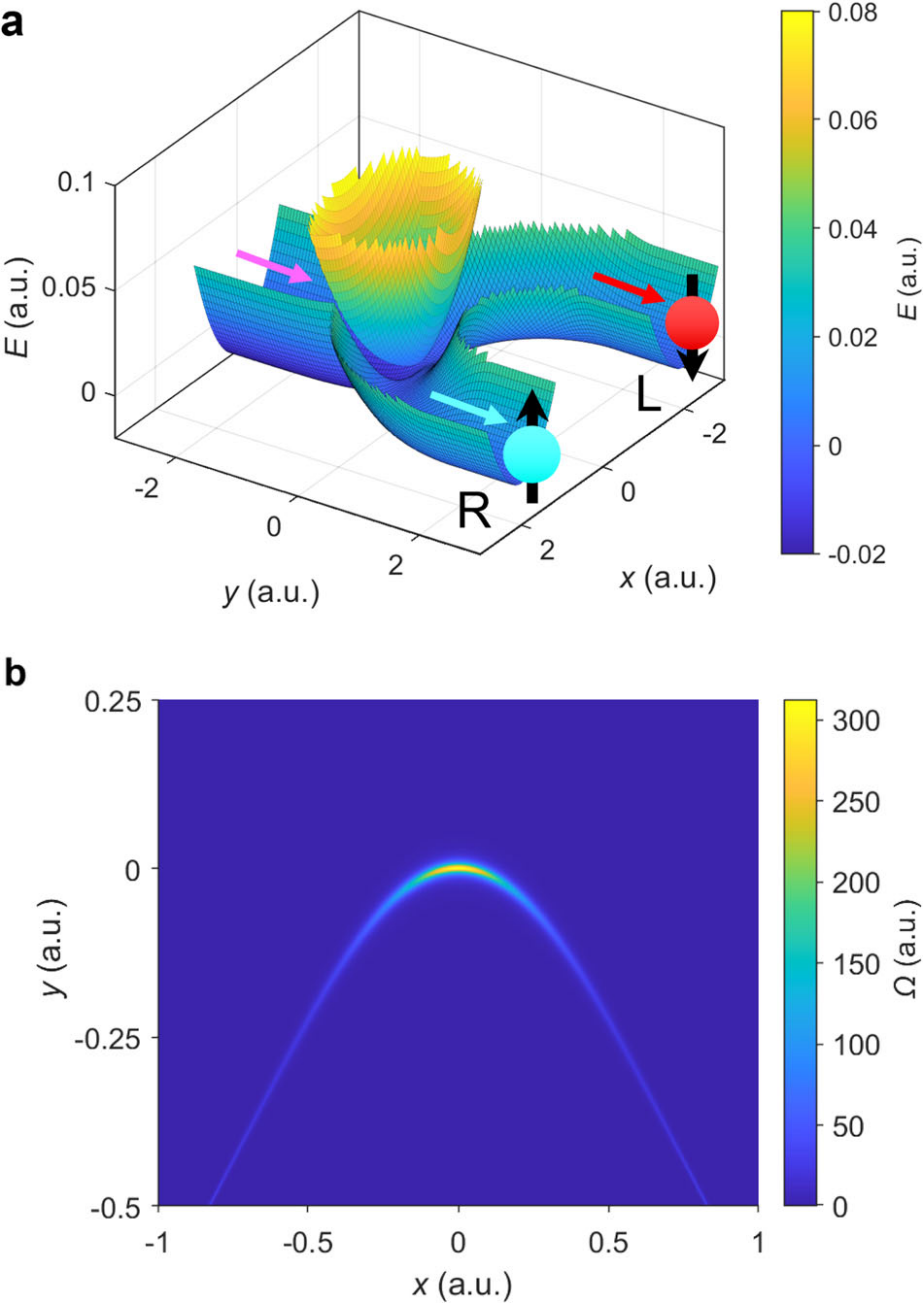
The potential surfaces and Berry curvature for the model Hamiltonian (Eq. (7)). **a** The adiabatic potential surfaces. On the ground state, the incoming channel (marked by the magenta arrow) is in the direction *y* → −*∞* at *x* = 0, the two outgoing channels (L and R, marked by the red and blue arrow according to their spin preferences) are in the direction *y* → +*∞* at *x* = ±*r*_0_. Nuclear wave packets associated with the spin-up electronic states prefer channel R while nuclear wave packets associated with spin-down electronic states prefer channel L. The perturbed CI is at *x* = 0, *y* = 0. **b** The Berry curvature Ω of the ground adiabatic surface around the intersection point. Here the model parameters (defined in Eq. (8)) are: *A* = 0.02, *ω* = 0.01, *M* = 10^3^, *ϵ*_1_ = 2.5, *ϵ*_2_ = 2.5, *r*_0_ = 2, *μ* = 10^−3^, *λ* = 2 × 10^−4^ (all in atomic units). Note the avoided CI becomes a true CI when *λ* = 0 (Supplementary Fig. 1).

In this article, we will work with the electronic Hamiltonian plotted in Fig. 1. Mathematically, for the case of spin up electrons, we assume there are two electronic states (both with the same spin) and two nuclear degrees of freedom, for which there is one incoming channel and two outgoing channels:

7 $$H=\left[\begin{array}{ll}{E}_{1}(x,y)&V(x,y)\\ V{(x,y)}^{* }&{E}_{2}(x,y)\end{array}\right]$$

where

8 $${E}_{1}(x,y)= \, A({e}^{{\epsilon }_{1}y}-1)+\frac{1}{2}M{\omega }^{2}{x}^{2} \\ {E}_{2}(x,y)= \, \left\{\begin{array}{ll}\frac{1}{2}M{\omega }^{2}{\left(\!\sqrt{{(y-{r}_{0})}^{2}+{x}^{2}}-{r}_{0}\!\right)}^{2},\hfill&y\,<\,{r}_{0}\\ \min \{\frac{1}{2}M{\omega }^{2}{(x-{r}_{0})}^{2},\frac{1}{2}M{\omega }^{2}{(x+{r}_{0})}^{2}\},&y\ge {r}_{0}\end{array}\right.\\ V(x,y)= \, (\mu x+i\lambda ){e}^{-{\epsilon }_{2}^{2}{y}^{2}}$$

where *x*, *y* are the nuclear coordinates. The adiabatic surfaces of *H* are plotted in Fig. 1. The real part of *V*(*x*, *y*) (which equals $\mu x{e}^{-{\epsilon }_{2}^{2}{y}^{2}}$) represents the diabatic coupling and the imaginary part *V*(*x*, *y*) (which equals $\lambda {e}^{-{\epsilon }_{2}^{2}{y}^{2}}$) represents the SOC. Note that, if we ignore the SOC component of the Hamiltonian, there is a CI at (0, 0), just before the bifurcation of the two channels. However, the CI is perturbed or moved away when we add in the SOC, which should be a common situation for molecules with spin. Note that, for this Hamiltonian, the phase variation parameter is $W=\frac{\partial }{\partial x}\arctan (\frac{\lambda }{\mu x})=-\frac{\mu \lambda }{{\mu }^{2}{x}^{2}+{\lambda }^{2}}$, which can be very large when $x\ll \frac{\lambda }{\mu }$, indicating that there could be a strong field effect in the vicinity of the origin.

Below, we will imagine a situation where a nuclear wavepacket approaches the avoided crossing from the *y* →−*∞* channel and then can emerge in one of the two *y* → +*∞* channels that are displaced in the *x*-direction: The left (L) channel flows along *x* = *r*_0_; the right (R) channel flows along *x* = −*r*_0_. See Fig. 1. For the Hamiltonian in Eq. (7), we will show that the wavepacket chooses one channel predominantly over the other. Physically, this choice of channel means that a nuclear wavepacket with one spin (say spin up) will emerge into one channel. By symmetry, incoming wave packets with other spin will have the exact same preference for the other channel (i.e., the channel at *x* = −*r*_0_). These spin-dependent nuclear wave packets are plotted heuristically in Fig. 1a.

In Fig. 1b, we plot the Berry curvature of the ground adiabatic state $\Omega =-i({d}_{01}^{x}{d}_{10}^{y}-{d}_{10}^{x}{d}_{01}^{y})$. By definition, the Berry force that nuclei feel along the ground state is $[{{F}_{{\rm{B}}}}_{x},{{F}_{{\rm{B}}}}_{y}]=\hbar \Omega /M[{p}_{y},-{p}_{x}]$. The Berry force of the ground-state surface is significant in a small region around the avoided CI. Therefore, one might indeed expect that nuclei will experience a strong force when passing through such an “avoided” complex-valued CI region and there will be a large difference in transmission between the outgoing terminals.

### Transmission probabilities

We have run scattering calculations for the Hamiltonian in Eq. (7) using the exact procedure outlined in refs. ^25^^,^^41^. We calculate the transmission rates *T*_L_ and *T*_R_ for each channel in Fig. 1. The nuclei enter asymptotically from the *y* → −*∞* channel (with spin up and nuclear motion bound to the ground state in the *x* direction); the nuclei can emerge in either the L or R channels. The total incoming energy *E* is defined by $E={p}_{y}^{2}/2M+{E}_{\text{bound}}={p}_{y}^{2}/2M+\hbar \omega /2-A$.

**Fig. 2:**
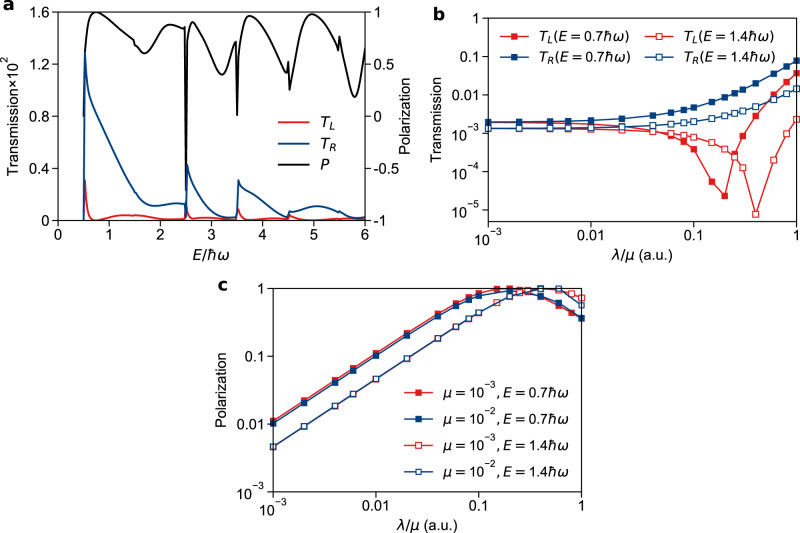
Transmission rates (*T*_L_ and *T*_R_) and spin selectivity *P* = (*T*_R_ − *T*_L_)/(*T*_R_ + *T*_L_) for dynamics along the Hamiltonian in Eq. (7). **a** *T*_L_, *T*_R_, and *P* as functions of the incoming energy. Note that the selectivity is very large, almost always >50% and often close to 100%. Here the model parameters (defined in Eq. (8)) are *λ* = 10^−3^ and *μ* = 2 × 10^−4^. **b** *T*_L_ and *T*_R_ as function of *λ*/*μ* with two fixed incoming energies *E* = 0.7$\hbar$*ω* and 1.4$\hbar$*ω* and *μ* = 10^−3^. While an increase in SOC (*λ*) always leads to an increase in *T*_R_, this monotonic behavior is not true for *T*_L_, leading to huge selectivity. **c** *P* as a function of *λ*/*μ* at incoming energies *E* = 0.7$\hbar$*ω*, 1.4$\hbar$*ω* and *μ* = 10^−3^, 10^−2^. Here *P* has little dependence on *μ* alone but depends on the reduced parameter *λ*/*μ*. The model parameters (defined in Eq. (8)) are: *A* = 0.02, *ω* = 0.01, *M* = 10^3^, *ϵ*_1_ = 2.5, *ϵ*_2_ = 2.5, *r*_0_ = 2 (all in atomic units). The source data can be found in Supplementary Information.

In Fig. 2a, we plot the individual transmission rates *T* for the two channels *T*_L_ and *T*_R_ as well as the spin selectivity *P* ≡ (*T*_R_ − *T*_L_)/(*T*_R_ + *T*_L_), both as functions of the total incoming energy *E*. Here one sees a huge preference for the right channel over almost all of the entire energy range. At certain incoming energies such as *E* = 0.7$\hbar$*ω*, 3.8$\hbar$*ω*, and 5.0$\hbar$*ω*, the selectivity is close to 1, such that nuclei with opposite electronic spins will be completely separated into the two outgoing channels. Note that, due to the quantized nature of the transverse bound states, one find peaks and valleys in the total transmission as a function of the incoming energy. Nevertheless, overall, this figure highlights the fact that an “avoided” complex-valued CI can produce enormous spin selection.

Next, in Fig. 2b, we plot *T*_L_ and *T*_R_ as a function of the reduced parameter *λ*/*μ* with a fixed *μ* = 10^−3^ at two incoming energies *E* = 0.7$\hbar$*ω* and 1.4$\hbar$*ω*. We find that *T*_R_ increases monotonically with *λ*/*μ* for most values of *λ*, but *T*_L_ actually decreases with *λ*/*μ* until a minimum is reached. Thus a complex-valued “avoided” CI can both promote some spin-dependent processes while suppressing others. Both of these effects will contribute to the total spin preference of the reaction.

Finally, in Fig. 2c, we plot the polarization *P* as a function of *λ*/*μ* with *μ* = 10^−3^  and 10^−2^, again with two incoming energies (*E* = 0.7$\hbar$*ω* and 1.4$\hbar$*ω*). Here we see that polarization changes very little with *μ* and depends (at least effectively) only on the reduced parameter *λ*/*μ*. As stated above, *P* has a maximum when *T*_L_ is minimized, and this maximum is basically unity (100% spin selectivity). We note that, even when *λ*/*μ* = 0.01, one can find a 10% preference for the right channel, which implies that even with a very small SOC, the presence of a Berry force around a complex-valued “avoided” CI can lead to significant spin effects.

## Discussion

It is now fully appreciated that a huge number of photochemical processes in solution are indeed mediated through CIs; even though the seams of CIs arise with co-dimension 2 in configuration space, the effects of the enormous derivative couplings around CIs fill up configuration space and act as a funnel to bridge different electronic surfaces. In the present article, we have shown that, whenever SOC is present, these CIs can also potentially lead to something not well appreciated: non-trivial spin polarization as mediated by a Berry force.

Looking forward, this article opens up many avenues for future experiments and theoretical investigation. First, there are many examples of photoinduced spin chemistry in the literature, for which time-dependent electron paramagnetic resonance spectroscopy can measure unpaired electrons interacting with their environment and for which there is no simple molecular explanation^42^. Furthermore, there are also hosts of magnetic field effects within organic photochemistry^24,43–45^ that have not yet been fully understood, including the hot topic nowadays of avian magnetoreception^44,46–48^. Overall, within the broad areas of organic photochemistry and spin chemistry, the impact of Berry forces has not yet been explored, which represents a huge opportunity for theoretical discovery.

Second, recent studies by Naaman et al. have shown that, even without photoexcitation or magnetic field effects, spin-dependent conductivity can arise when current is passed through chiral molecules, an effect known as CISS^21–23,49,50^. To date, theory has been unable to explain why the CISS effect is as large as it is, given how small the SOC matrix elements are^22,50–55^. The present article must make one wonder whether CIs can be found within the manifold of conducting electronic states, such that a Berry force can help explain the large magnitude of spin selection. To our knowledge, no one has (as of yet) looked for an isotope effect within the context of CISS experiments, but such an isotope effect would indeed confirm such a hypothesis. We note that recent studies have shown that electron transfer in organic molecules such as DNA and proteins are largely incoherent^56–59^, which is consistent with the possibility that nuclear motion (and the associated Berry force) may well lead to spin selectivity. Moreover, the CISS effect has already been shown to lead to changes in overpotential for water splitting and novel magnetic field effects^60^, suggesting that, if we can indeed use Berry force to produce spin-selected molecular fragments, there may be a host of future applications, including new spin-dependent catalytic mechanisms, exotic stable molecular spin devices, and efficient electrochemical metal–ion separation protocols.

Now, the experiments above represent interesting potential applications in spin chemistry and physics. At the same time, however, we must emphasize that, in order for practical progress to be made with quantitative experimental predictions, two theoretical questions will need to be addressed. First, in the present article, we have used exact quantum mechanics to calculated scattering rates. These calculations are very expensive and do not always offer a simple explanation of the physics we observe. More generally, for large systems, we will require new semiclassical tools (that treat nuclei classically) that are both inexpensive and that can offer intuitive pictures of electronic and spin relaxation. Developing such tools (e.g., extending Tully’s surface hopping algorithm^29,61^ to the case of complex-valued Hamiltonians) will be essential if we are to study systems with many electronic states and many nuclear degrees of freedom (ideally ab initio systems).

Second, the question of exactly when and how Berry force and/or magnetic fields lead to observable effects in the condensed phase remains a general problem for spin chemistry. On the one hand, from a classical perspective, a magnetic field does not affect the equilibrium solution to a Fokker–Planck equation, and the magnitude of molecular SOC or hyperfine interactions are orders of magnitude smaller than the thermal energy *k*_B_*T* in the room temperature^43,50^. Thus one might be led to believe that magnetic field effects (and thus Berry force) must vanish with enough friction. On the other hand, however, the Berry force near a complex-valued avoided CI can be very large. Furthermore, for a molecule that is exposed to an out-of-equilibrium environment (e.g., a current running through the molecule), the fluctuation-dissipation theorem does not hold and there is no reason to expect that external friction will eliminate the influence of Berry force and/or spin polarization. For example, a molecule near a metal surface will feel a Berry force in the form of an asymmetric electronic friction tensor when there is an electric current^62–67^. Note that Naaman and other researchers have shown that the CISS effect increases with increasing voltage, such that the molecular dynamics is far from equilibrium^23,68–71^. Obviously, extrapolating from the present simulations to the condensed phase, and calculating the effect of a Berry-force induced spin polarization in the presence of friction, will be a crucial next step forward. Clearly, several key obstacles remain if we are to ever merge theoretical chemistry with the field of spintronics.

In summary, we have demonstrated that dynamics in the vicinity of a complex-valued avoided CI can lead to extremely strong spin selectivity for reaction pathways (close to 100%); this selectivity can hold even when the SOC matrix elements are weak. This article suggests that, in the future, simulations of nonadiabatic dynamics through CIs may find enormous spin polarization effects if SOC is included and Berry force is taken into account. Furthermore, in practice, this article also highlights the possibility that, with a proper understanding of photochemical mechanisms, organic chemists may be able to synthesize molecules that ensure spin selection, thus taking a very different approach toward the development of spintronics.

## Supplementary information

Supplementary Information

## Data Availability

The authors declare that the data supporting the findings of this study are available within the paper and the supplementary information files. Source data are provided with this paper.

## Source data

Source Data

## Code availability

The code used for scattering calculation is available at https://github.com/subotnikgroup/scatter2^72^.

## References

[1] Žutić I, Fabian J, Sarma SD (2004). Spintronics: fundamentals and applications. Rev. Mod. Phys..

[2] Rocha AR (2005). Towards molecular spintronics. Nat. Mater..

[3] Fert A (2008). Origin, development, and future of spintronics (Nobel lecture). Angew. Chem. Int. Ed..

[4] Chumak AV, Vasyuchka VI, Serga AA, Hillebrands B (2015). Magnon spintronics. Nat. Phys..

[5] Linder J, Robinson JW (2015). Superconducting spintronics. Nat. Phys..

[6] Jungwirth T, Marti X, Wadley P, Wunderlich J (2016). Antiferromagnetic spintronics. Nat. Nanotechnol..

[7] Baltz V (2018). Antiferromagnetic spintronics. Rev. Mod. Phys..

[8] Baibich MN (1988). Giant magnetoresistance of (001)Fe/(001)Cr magnetic superlattices. Phys. Rev. Lett..

[9] Moodera JS, Kinder LR, Wong TM, Meservey R (1995). Large magnetoresistance at room temperature in ferromagnetic thin film tunnel junctions. Phys. Rev. Lett..

[10] Gambardella P, Miron IM (2011). Current-induced spin-orbit torques. Philos. Trans. R. Soc. A Math. Phys. Eng. Sci..

[11] Brataas A, Kent AD, Ohno H (2012). Current-induced torques in magnetic materials. Nat. Mater..

[12] Manchon A (2019). Current-induced spin-orbit torques in ferromagnetic and antiferromagnetic systems. Rev. Mod. Phys..

[13] Berger L (1996). Emission of spin waves by a magnetic multilayer traversed by a current. Phys. Rev. B Condens. Matter Mater. Phys..

[14] Slonczewski JC (1996). Current-driven excitation of magnetic multilayers. J. Magn. Magn. Mater..

[15] Ralph DC, Stiles MD (2008). Spin transfer torques. J. Magn. Magn. Mater..

[16] Mahfouzi F, Nagaosa N, Nikolić BK (2012). Spin-orbit coupling induced spin-transfer torque and current polarization in topological-insulator/ferromagnet vertical heterostructures. Phys. Rev. Lett..

[17] Bajpai U, Nikolić BK (2019). Time-retarded damping and magnetic inertia in the Landau-Lifshitz-Gilbert equation self-consistently coupled to electronic time-dependent nonequilibrium Green functions. Phys. Rev. B.

[18] Hirsch JE (1999). Spin hall effect. Phys. Rev. Lett..

[19] Nikolić BK, Souma S, Zĝrbo LP, Sinova J (2005). Nonequilibrium spin hall accumulation in ballistic semiconductor nanostructures. Phys. Rev. Lett..

[20] Sinova J, Valenzuela SO, Wunderlich J, Back CH, Jungwirth T (2015). Spin Hall effects. Rev. Mod. Phys..

[21] Naaman R, Waldeck DH (2012). Chiral-induced spin selectivity effect. J. Phys. Chem. Lett..

[22] Naaman R, Waldeck DH (2015). Spintronics and chirality: spin selectivity in electron transport through chiral molecules. Annu. Rev. Phys. Chem..

[23] Naaman R, Paltiel Y, Waldeck DH (2020). Chiral molecules and the spin selectivity effect. J. Phys. Chem. Lett..

[24] Gould IR, Turro NJ, Zimmt MB (1984). Magnetic field and magnetic isotope effects on the products of organic reactions. Adv. Phys. Org. Chem..

[25] Wu Y, Miao G, Subotnik JE (2020). Chemical reaction rates for systems with spin-orbit coupling and an odd number of electrons: does Berry’s phase lead to meaningful spin-dependent nuclear dynamics for a two state crossing?. J. Phys. Chem. A.

[26] Berry MV (1984). Quantal phase factors accompanying adiabatic changes. Proc. R. Soc. Lond. A. Math. Phys. Sci..

[27] Takatsuka K, Yonehara T (2011). Exploring dynamical electron theory beyond the Born-Oppenheimer framework: from chemical reactivity to non-adiabatically coupled electronic and nuclear wavepackets on-the-fly under laser field. Phys. Chem. Chem. Phys..

[28] Berry MV, Robbins JM (1993). Chaotic classical and half-classical adiabatic reactions: geometric magnetism and deterministic friction. Proc. R. Soc. Lond. Ser. A Math. Phys. Sci..

[29] Tully JC (1990). Molecular dynamics with electronic transitions. J. Chem. Phys..

[30] Juanes-Marcos JC, Althorpe SC, Wrede E (2005). Theoretical study of geometric phase effects in the hydrogen-exchange reaction. Science.

[31] Yarkony DR (1996). Diabolical conical intersections. Rev. Mod. Phys..

[32] Guo H, Yarkony DR (2016). Accurate nonadiabatic dynamics. Phys. Chem. Chem. Phys..

[33] Xie C, Malbon CL, Yarkony DR, Xie D, Guo H (2018). Signatures of a conical intersection in adiabatic dissociation on the ground electronic state. J. Am. Chem. Soc..

[34] Matsika S, Yarkony DR (2001). On the effects of spin-orbit coupling on conical intersection seams in molecules with an odd number of electrons. II. Characterizing the local topography of the seam. J. Chem. Phys..

[35] Matsika S, Yarkony DR (2001). On the effects of spin-orbit coupling on conical intersection seams in molecules with an odd number of electrons. I. Locating the seam. J. Chem. Phys..

[36] Matsika S, Yarkony DR (2002). Spin-orbit coupling and conical intersections in molecules with an odd number of electrons. III. A perturbative determination of the electronic energies, derivative couplings and a rigorous diabatic representation near a conical intersection. J. Chem. Phys..

[37] Matsika S, Yarkony DR (2002). Spin-orbit coupling and conical intersections. IV. A perturbative determination of the electronic energies, derivative couplings, and a rigorous diabatic representation near a conical intersection. The general case. J. Phys. Chem. B.

[38] Truhlar DG, Mead CA (2003). Relative likelihood of encountering conical intersections and avoided intersections on the potential energy surfaces of polyatomic molecules. Phys. Rev. A.

[39] Truhlar DG, Mead CA (2011). Comment on “optical conversion of conical intersection to avoided crossing” by Y. Arasaki and K. Takatsuka, Phys. Chem. Chem. Phys., 2010, 12, 1239. Phys. Chem. Chem. Phys..

[40] Yang B, Gagliardi L, Truhlar DG (2018). Transition states of spin-forbidden reactions. Phys. Chem. Chem. Phys..

[41] Ouyang W, Dou W, Subotnik JE (2015). Surface hopping with a manifold of electronic states. I. Incorporating surface-leaking to capture lifetimes. J. Chem. Phys..

[42] Hoff, A. J. *Advanced EPR: Applications in Biology and Biochemistry* (Elsevier, 2012).

[43] Steiner UE, Ulrich T (1989). Magnetic field effects in chemical kinetics and related phenomena. Chem. Rev..

[44] Hore PJ, Mouritsen H (2016). The radical-pair mechanism of magnetoreception. Annu. Rev. Biophys..

[45] Hore PJ, Ivanov KL, Wasielewski MR (2020). Spin chemistry. J. Chem. Phys..

[46] Hiscock HG (2016). The quantum needle of the avian magnetic compass. Proc. Natl Acad. Sci. USA.

[47] Mouritsen H (2018). Long-distance navigation and magnetoreception in migratory animals. Nature.

[48] Fay TP, Lindoy LP, Manolopoulos DE (2018). Spin-selective electron transfer reactions of radical pairs: beyond the Haberkorn master equation. J. Chem. Phys..

[49] Göhler B (2011). Spin selectivity in electron transmission through self-assembled monolayers of double-stranded DNA. Science.

[50] Naaman R, Paltiel Y, Waldeck DH (2019). Chiral molecules and the electron spin. Nat. Rev. Chem..

[51] Varela S, Mujica V, Medina E (2016). Effective spin-orbit couplings in an analytical tight-binding model of DNA: spin filtering and chiral spin transport. Phys. Rev. B.

[52] Maslyuk VV, Gutierrez R, Dianat A, Mujica V, Cuniberti G (2018). Enhanced magnetoresistance in chiral molecular junctions. J. Phys. Chem. Lett..

[53] Zöllner MS, Varela S, Medina E, Mujica V, Herrmann C (2020). Insight into the origin of chiral-induced spin selectivity from a symmetry analysis of electronic transmission. J. Chem. Theory Comput..

[54] Zöllner MS, Mujica V, Herrmann C (2020). The influence of electronic structure modelling and junction structure on first-principles chiral induced spin selectivity. J. Chem. Theory Comput..

[55] Evers F, Korytár R, Tewari S, van Ruitenbeek JM (2019). Advances and challenges in single-molecule electron transport. Rev. Mod. Phys..

[56] Zhang Y, Liu C, Balaeff A, Skourtis SS, Beratan DN (2014). Biological charge transfer via flickering resonance. Proc. Natl Acad. Sci. USA.

[57] Xiang L (2015). Intermediate tunnelling-hopping regime in DNA charge transport. Nat. Chem..

[58] Kim H, Kilgour M, Segal D (2016). Intermediate coherent-incoherent charge transport: DNA as a case study. J. Phys. Chem. C.

[59] Beratan DN (2019). Why are DNA and protein electron transfer so different?. Annu. Rev. Phys. Chem..

[60] Zhang W, Banerjee-Ghosh K, Tassinari F, Naaman R (2018). Enhanced electrochemical water splitting with chiral molecule-coated Fe3O4 nanoparticles. ACS Energy Lett..

[61] Coker DF, Xiao L (1995). Methods for molecular dynamics with nonadiabatic transitions. J. Chem. Phys..

[62] Lü J-T, Brandbyge M, Hedegård P, Todorov TN, Dundas D (2012). Current-induced atomic dynamics, instabilities, and Raman signals: quasiclassical Langevin equation approach. Phys. Rev. B.

[63] Bode N, Kusminskiy SV, Egger R, von Oppen F (2012). Current-induced forces in mesoscopic systems: a scattering-matrix approach. Beilstein J. Nanotechnol..

[64] Thomas M, Karzig T, Kusminskiy SV, Zaránd G, Von Oppen F (2012). Scattering theory of adiabatic reaction forces due to out-of-equilibrium quantum environments. Phys. Rev. B.

[65] Dzhioev AA, Kosov DS, Von Oppen F (2013). Out-of-equilibrium catalysis of chemical reactions by electronic tunnel currents. J. Chem. Phys..

[66] Dou W, Subotnik JE (2018). Perspective: How to understand electronic friction. J. Chem. Phys..

[67] Dou W, Subotnik JE (2018). Universality of electronic friction. II. Equivalence of the quantum-classical Liouville equation approach with von Oppen’s nonequilibrium Green’s function methods out of equilibrium. Phys. Rev. B.

[68] Xie Z (2011). Spin specific electron conduction through DNA oligomers. Nano Lett..

[69] Kettner M (2015). Spin filtering in electron transport through chiral oligopeptides. J. Phys. Chem. C..

[70] Kiran V (2016). Helicenes - a new class of organic spin filter. Adv. Mater..

[71] Kettner M (2018). Chirality-dependent electron spin filtering by molecular monolayers of helicenes. J. Phys. Chem. Lett..

[72] Wu, Y. & Subotnik, J. E. Electronic spin separation induced by nuclear motion near conical intersections. Scatter2. 10.5281/zenodo.4299303 (2020). 10.1038/s41467-020-20831-8 PMC7846775 33514700

