# Supplementary material for: Electronic spin separation induced by nuclear motion near conical intersections
Source: Nat Commun. 2021 Jan 29;12:700. doi: 10.1038/s41467-020-20831-8 (PMC7846775; doi:10.1038/s41467-020-20831-8)
Supplement: Supplementary file 1 — Supplementary Information [file 41467_2020_20831_MOESM1_ESM.pdf]

Supplementary Information for "Molecular Motion and Electronic Spin Separation: Do We Fully Understand Conical Intersections?"

Yanze Wu[1], Joseph E. Subotnik[1]

[1]*Department of Chemistry, University of Pennsylvania, Philadelphia, Pennsylvania 19104, USA*

1. Energy surface of model system with a true conical intersection

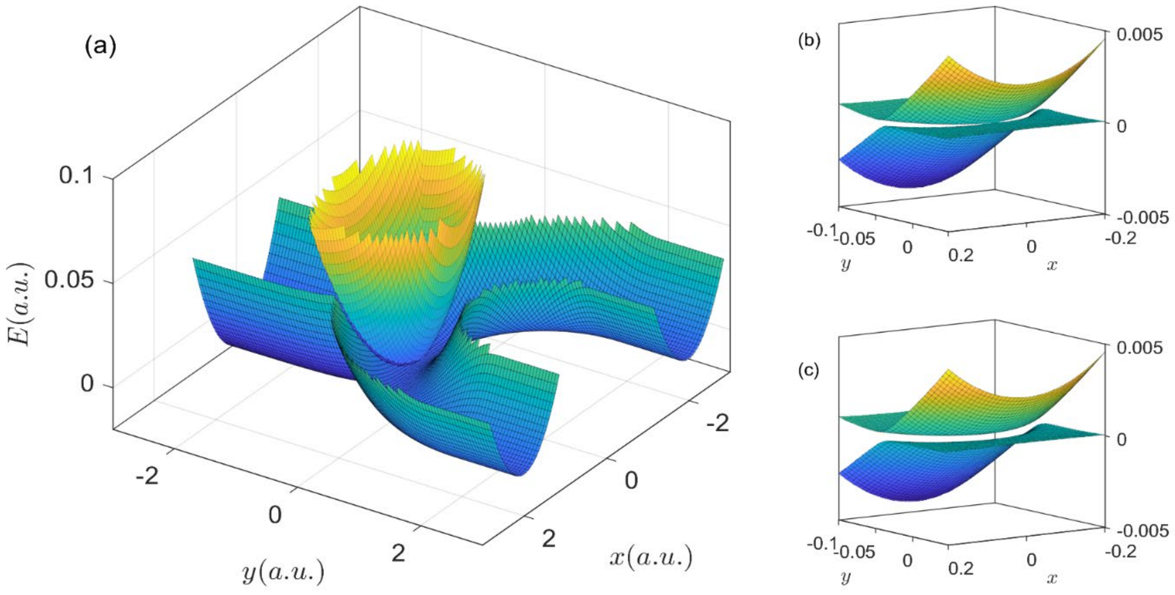

Supplementary Figure 1 (a) Adiabatic energy surface of Hamiltonian (7) with $\lambda = 0$, where a conical intersection lies in (0,0). (b) (c) Comparison of adiabatic surfaces near the CI. In (b) $\lambda = 0$ and in (c) $\lambda = 2 \times 10^{-4}$. The other parameters are $A = 0.02$, $\omega = 0.01$, $M = 10^3$, $\epsilon_1 = \epsilon_2 = 2.5$, $r_0 = 2$, $\mu = 10^{-3}$.
